# A national outbreak of *Serratia marcescens* complex: investigation reveals genomic population structure but no source, Norway, June 2021 to February 2023

**DOI:** 10.2807/1560-7917.ES.2025.30.5.2400291

**Published:** 2025-02-06

**Authors:** Arne Michael Taxt, Vegard Eldholm, Nicola Isabelle Kols, Maria Schei Haugan, Niclas Raffelsberger, Anne Mette Asfeldt, André Ingebretsen, Anita Blomfeldt, Kristin Stenhaug Kilhus, Paul Christoffer Lindemann, Horst Bentele, Jeanette Stålcrantz, Liz Ertzeid Ødeskaug, Thale Cathrine Berg, The Norwegian Serratia study group, The Norwegian Serratia study group, Siv Anita Kvaal, Lene Christin Olsen, Torunn Gresdal Rønning, Anette Skjærvik, Randi Solheim, Christina Gabrielsen Ås, Iren Høyland Löhr, Kirsten Gravningen, Silje Severine Sætre, Karin Helmersen, Anne K Steffensen, Stephan A Frye, Jan Cato Holter, Hanne-Merete Eriksen-Volle, Bjørn Iversen, Turid M. Berglund, Rune Jemtland, Raymond Ludvigsen, Bodil Weng, Torunn Annie Pedersen, Anna Kaarina Pöntinen

**Affiliations:** 1 Norwegian Institute of Public Health, Oslo, Norway; 2 ECDC fellowship Programme, Public Health Microbiology path (EUPHEM), European Centre for Disease Prevention and Control (ECDC), Solna, Sweden; 3 Regional Centre for Infection Prevention and Control, Central Norway Regional Health Authority, St. Olavs Hospital, Trondheim University Hospital, Trondheim, Norway; 4 Department of Medical Microbiology, St. Olavs Hospital, Trondheim University Hospital, Trondheim, Norway; 5 Department of Microbiology and Infection Control, University Hospital of North Norway, Tromsø, Norway; 6 Regional Centre for Infection Control, Northern Norway Regional Health Authority, Department of Microbiology and Infection Control, University Hospital of North Norway, Tromsø, Norway; 7 Department of Community Medicine, UiT, The Arctic University of Norway, Tromsø, Norway; 8 Department of Microbiology, Oslo University Hospital, Oslo, Norway; 9 Department of Infection Prevention, Oslo University Hospital, Oslo, Norway; 10 Department of Microbiology and Infection Control, Akershus University Hospital, Lørenskog, Norway; 11 Western Norway Advisory Unit on IPC, Department of Research and Development, Haukeland University Hospital, Bergen, Norway; 12 Department of Microbiology, Haukeland University Hospital, Bergen, Norway; 13 The members of The Norwegian Serratia study group are listed under collaborators

**Keywords:** outbreak, Serratia marcescens, whole genome sequencing, WGS, clonality, unknown source, ct755

## Abstract

We report a national outbreak of *Serratia marcescens* complex type 755 (ct755) in Norway, with 74 cases identified between June 2021 and February 2023. Careful reviews of patient journals and interviews were performed, involving 33 hospitals throughout Norway. All available clinical isolates of *S. marcescens* collected between January 2021 and February 2023 (n = 455, including cases) from all involved hospitals were whole genome sequenced. Cases displayed a pattern of opportunistic infections, as usually observed with *S. marcescens*. No epidemiological links, common exposures or common risk factors were identified. The investigation pointed to an outbreak source present in the community. We suspect a nationally distributed product, possibly a food product, as the source. Phylogenetic analysis revealed a highly diverse bacterial population containing multiple distinct clusters. The outbreak cluster ct755 stands out as the largest and least diverse clone of a continuum, however a second cluster (ct281) also triggered a separate outbreak investigation. This report highlights challenges in the investigation of outbreaks caused by opportunistic pathogens and suggests that the presence of identical strains of *S. marcescens* in clinical samples is more common than previously recognised.

**Key public health message**

**What did you want to address in this study and why?**

We report an outbreak in Norway of the opportunistic pathogen *Serratia marcescens*, which causes infections primarily in people with weakened immune systems and intensive-care patients. The outbreak initially appeared to be local and hospital-associated, however an extensive investigation revealed a large national outbreak that also involved the community.

**What have we learnt from this study?**

The presence of identical strains of* Serratia marcescens* in clinical samples from different patients is more common than previously recognised. This is only revealed when bacterial isolates are characterised in detail by whole genome sequencing. Outbreak investigations of opportunistic pathogens require a multidisciplinary approach including microbiological and bioinformatic expertise.

**What are the implications of your findings for public health?**

During an outbreak with an opportunistic pathogen, investigating and/or ruling out a possible hospital-associated outbreak is a key first step. Finding a definitive community source, however, can be extremely challenging and may sometimes not be feasible.

## Background

The *Serratia marcescens* complex (SMC) encompasses a number of closely related species, until recently referred to simply as ‘*Serratia marcescens*’ [1-4]. They are Gram-negative rod-shaped bacteria in the order *Enterobacterales*. First described in 1819, *S. marcescens* was considered non-pathogenic until the 1970s when its clinical significance as an opportunistic pathogen was recognised [5]. Asymptomatic colonisation is well documented and generally believed to be transient, although persistent colonisation with a single strain of *S. marcescens* for more than a year has been reported [6,7]. The bacteria tend to form biofilms and are capable of surviving on dry inanimate surfaces for weeks to months [5,8]. Persistence in hospital sinks for years has recently been described [1]. Distribution of SMC in the environment is considered ubiquitous, and it has been isolated from soil, water, animals, plants and farming environments. The SMC is a known cause of infections in animals, and particularly well documented is its role as a pathogen in insects and as a cause of mastitis in cattle [4,9].

Numerous nosocomial outbreaks have been reported, typically in neonatal wards or intensive care units, but also involving multiple wards and more than one hospital [10]. Typically, local nosocomial outbreaks are resolved by reinforcing basic infection control measures, and a defined source of the outbreak is often not identified. Large and geographically widespread nosocomial outbreaks linked to contaminated medical products have also been reported [11].

We here report an outbreak of SMC in Norway that initially presented as a nosocomial outbreak but after an extensive investigation proved to be much more widespread temporally and geographically than initially perceived, and also involved the community.

## Outbreak detection

The outbreak was first reported to the Norwegian Institute of Public Health (NIPH) by the Central Norway Regional Health Authority on 11 October 2022. Seven SMC isolates from clinical blood cultures taken between May and September 2022 had been subjected to whole genome sequencing (WGS) and found to belong to the same clone. It received a novel core genome multilocus sequence typing (cgMLST) designation as complex type 755 (ct755) [12]. The strain was non-pigmented and displayed a sensitive antibiotic susceptibility pattern, including susceptibility to all beta-lactams. The cases were from four different hospitals, and local investigations had not identified any epidemiological links between cases. A national outbreak was recognised on 3 November 2022 when three additional cases of ct755 were reported from the south-eastern Norway Regional Health Authority. A national outbreak investigation team with participants from all four Regional Health Authorities, the Norwegian Food Safety Authority and The Norwegian Medicines Agency was established under the coordination of NIPH.

The initial suspicion was that a common source, possibly present in the hospital environment, was causing the outbreak. Primary objectives of the outbreak investigation were to characterise the outbreak, identify its source, and ultimately contain transmission. A secondary objective was later established: to rule out a nosocomial source.

During the outbreak investigation of ct755 it became evident from genomic analysis that additional clusters of SMC also were present in multiple clinical samples with diverse geographical and temporal distribution. This outbreak report focuses on ct755 but includes some observations and analyses relating to other SMC clusters, in particular ct281, to provide a broader context. The ct281 cluster was subject to a parallel national outbreak investigation, however, on a more limited scale than ct755.

## Methods

### Case definition and case finding

Retrospective case-finding was conducted by all Norwegian clinical microbiological laboratories, and WGS was applied to all available stored clinical isolates (mainly blood cultures) of SMC dating back to 1 January 2021. Prospectively, all clinical isolates of SMC were screened by either WGS or a clone-specific PCR developed at the Department of Medical Microbiology, St. Olavs hospital, Trondheim University Hospital for the investigation (see the Supplement for details). We defined a confirmed case as a person who tested positive for SMC ct755 after 1 January 2021.

### Epidemiological investigation

We recorded detailed information about patient demographics, mortality, risk factors, exposures and recent hospital admissions. Local infection control personnel collected data from hospital records, including medical procedures, medicines and use of invasive devices. When possible, cases who did not have recent hospital admissions were interviewed to collect data on exposures and possible sources of infection outside the hospital setting. We developed a questionnaire that covered community exposures including use of medical products, cosmetic products and basic food-related questions. All data were analysed at the national level by NIPH using Stata 17.0 and MS Office Excel.

### International inquiry

An inquiry with information about the outbreak and details about the outbreak strain was submitted on 11 November 2022 through the European surveillance portal for infectious diseases (EpiPulse). The information was updated on 25 January 2023.

### Sequence generation

The regional clinical microbiological laboratories performed WGS of SMC isolates and shared the data with NIPH on a secure project site set up at the Norwegian e-Infrastructure for Life Sciences. The sequencing data were generated on three different platforms: Illumina, Oxford Nanopore and IonTorrent. Sequencing data from 455 isolates of SMC were included in the analyses after exclusion of data not meeting quality thresholds (see following section). We used SeqSphere [13] to assign isolates to clonal complexes with the appropriate cgMLST scheme [12]. Raw sequencing reads are available under ENA project accession PRJEB62502.

### Sequence assembly and phylogenetic analyses

Collective analysis of data from all sequencing laboratories was performed at NIPH. Raw reads generated on the Illumina platform were trimmed using Trimmomatic v0.39 [14]. Illumina and IonTorrent reads were subsequently assembled de novo using SPAdes v3.13.0 [15] with ‘careful’ mode and automatic coverage cut-off. The IonTorrent option was specified for IonTorrent reads. Oxford Nanopore long reads were assembled using Flye v2.9.1-b1780 [16] with the ‘nano-hq’ option. Quast [17] was used for quality control of the assemblies. Due to the high diversity in the genome dataset, we did not apply a priori cut-offs for the exclusion of assemblies but rather looked for outliers. Assemblies contained between 4.9 and 5.8 million nt, assembled into a median of 25 (1–400) contigs. A single isolate was assembled into 7.2 million nt and was excluded on this basis. The median sequencing depth was 58× (10× – 252×).

On 10 November 2022, soon after the outbreak of ct755 had been recognised as a national outbreak, we downloaded all *S. marcescens* genomes available in the National Center for Biotechnology Information (NCBI) assembly database (n = 1,547) to identify the closest sequenced relatives of ct755 and ct281, the two largest complex types identified in Norway. Mash v.2.3 [18] was used to rapidly screen and identify the closest relative of each complex type. Assemblies downloaded from NCBI were not subjected to quality control measures as they were not used beyond contextualisation of the Norwegian isolates. For assignment of species, we assigned all isolates to the closest matching genome in the Genome Taxonomy Database (GTDB) [19] using the ‘ani_rep’ function in gtdb-tk [20].

We used PopPUNK v2.5.0 [21] to perform kmer-based clustering and phylogenetic analyses on a weekly basis throughout the outbreak investigation (not shown). As the sequencing data were generated on three different platforms and not easily integrated in a single workflow for reference-based mapping, single nucleotide polymorphisms (SNPs) were called for all isolates by aligning assemblies to a reference (GCF_003516165_1_ASM351616v1) using Snippy v4.6.0 (https://github.com/tseemann/snippy ) with the ctgs option. The reference genome was chosen on the basis of being a publicly available high-quality closed genome assembled using both short and long read sequences. Core-genome SNP alignments were subsequently generated with snippy-core. A maximum likelihood phylogenetic tree was generated using FastTree v.2.1.11 [22] and visualised in Microreact [23].

### Temporal phylogenetic analyses

As part of an independent analysis of ct755, SNPs were called from raw sequencing reads for isolates belonging to this complex type. Due to higher error rate, we excluded isolates sequenced on the Oxford Nanopore platform. Raw reads were mapped to the assembly representing the first identified ct755 case (SO_2022_08_22_013) with ‘minfrac’ set to 0.9 and ‘mincov’ to 8. The minimum sequencing depth for ct755 samples was 38×. A core genome alignment was generated with snippy-core. SNP-dists (https://github.com/tseemann/snp-dists) was used to output pairwise SNP distances within the complex. The full alignment was used to generate a maximum likelihood tree with IQ-TREE [24] with HKY + F + I identified as the best model.

We performed a temporal phylogenetic analysis using TreeTime v.0.9.4 [25] with the ’confidence’ and ’covariation‘ options. The full core alignment from Snippy, a date file summarising the sampling dates for each sequence, and the phylogenetic tree from IQ-TREE were used as inputs.

## Results

### Overall diversity of *Serratia marcescens* complex isolates

**Figure 1:**
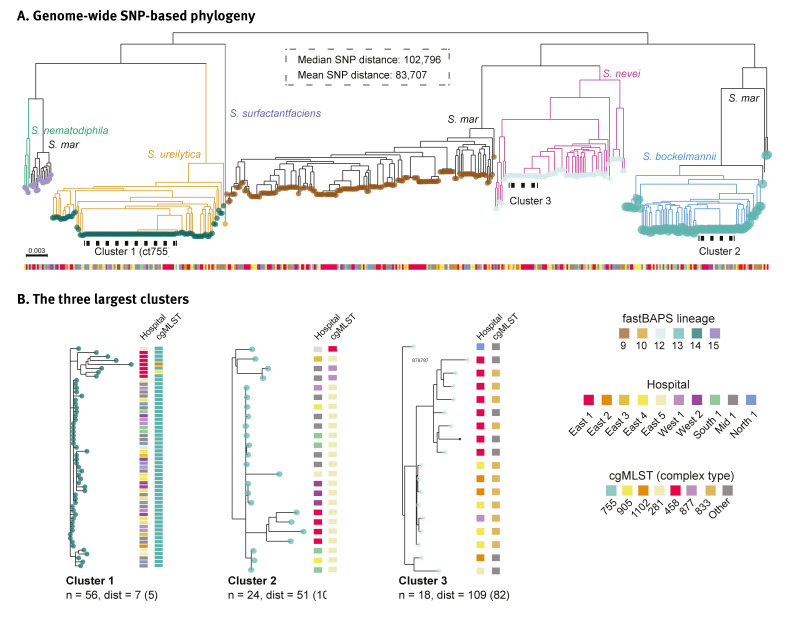
Phylogenetic analyses of *Serratia marcescens* clinical isolates showing the three major clusters, Norway, June 2021 to February 2023 (n = 455)

A total of 455 SMC isolates were sequenced as part of the outbreak investigation, distributed across six SMC species as follows: *S. marcescens* (n = 181), *S. ureilytica* (n = 105), *S. bockelmannii* (n = 84), *S. nevei* (n = 80), *S. nematodiphila* (n = 4) and *S. surfactantfaciens* (n = 1). Phylogenetic analyses and species assignments based on nucleotide identity against the GTDB were largely concordant: Apart from *S. marcescens*, the different species formed monophyletic clades and were assigned to unique fastBAPS lineages (Figure 1). The 181 *S. marcescens* isolates on the other hand were split between three different clades/lineages, with the vast majority belonging to fastBAPS lineage 9. The ct755 isolates belonged to *S. ureilytica* (fastBAPS lineage 14), whereas ct281 isolates were identified as *S. bockelmannii* (fastBAPS lineage 13).

cgMLST: core genome multilocus sequencing typing; dist: median pairwise distance;* S. mar*: *S. marcescens*; SNP: single nucleotide polymorphism.

Panel A: Tree built from assemblies using Snippy and FastTree. A total of 455 Norwegian SMC genomes are included, plus two context genomes representing the closest identified sequence identified on NCBI assembly for each of ct755 and ct281. Tree branches are coloured by species. Panel B: Detailed view of the three largest clusters annotated with hospital and cgMLST type. The estimated median pairwise distance for all samples within a cluster is given below each phylogeny (dist = N). Numbers in parentheses indicate pairwise distances after exclusion of samples sequenced on the Oxford Nanopore platform. In both panels A and B the sample tips are coloured by fastBAPS lineage as defined by Williams et al. [4].

### Descriptive epidemiology of outbreak strain ct755 (*Serratia ureilytica*)

**Figure 2:**
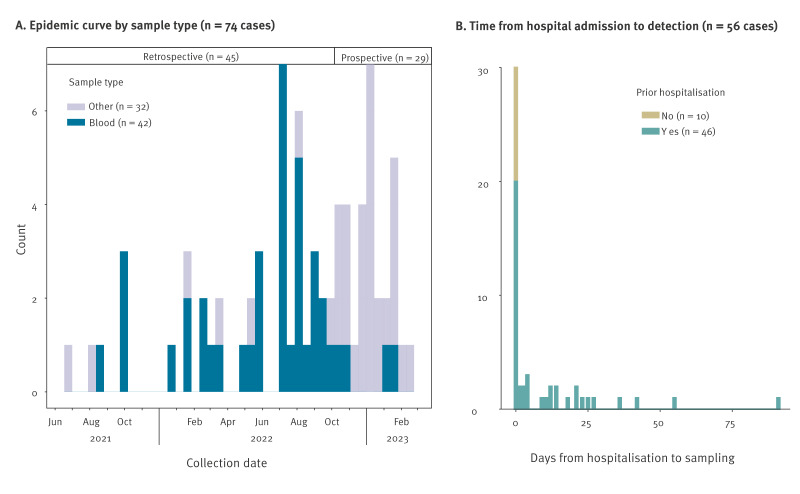
Overview of *Serratia marcescens* complex ct755 epidemiological parameters, Norway, June 2021 to February 2023

We identified 74 cases with SMC ct755 between June 2021 and February 2023 in 33 hospitals in all four Norwegian health regions (Figure 2A). Among those, 66 cases had ct755 detected in one sample material only, whereas eight cases had ct755 detected in more than one sample material. The outbreak strain was detected most commonly in blood cultures (n = 42), followed by urine (n = 15), respiratory samples (n = 7), wound samples (n = 6) and other samples (n = 14). Among the retrospectively sequenced isolates blood culture as material was overrepresented since most laboratory guidelines dictate storage of pathogens cultivated from sterile compartments, while isolates from non-sterile samples are discarded.

Panel A: Only one sample per case is included in the curve and shown by date of first positive sample, indicated either as positive blood culture or, if no blood culture was available, as other sample material. Panel B: The date of admission for the hospital stay during which the outbreak strain was detected was available for 56 cases. For 30 of these the outbreak strain was detected in a sample collected on the day of admission (day 0). Eleven of these 30 cases had been hospitalised in the 4 weeks before admission, nine had been hospitalised but more than 4 weeks before admission. Six of the 10 patients without previous history of hospitalisation had visited an outpatient clinic.

**Figure 3:**
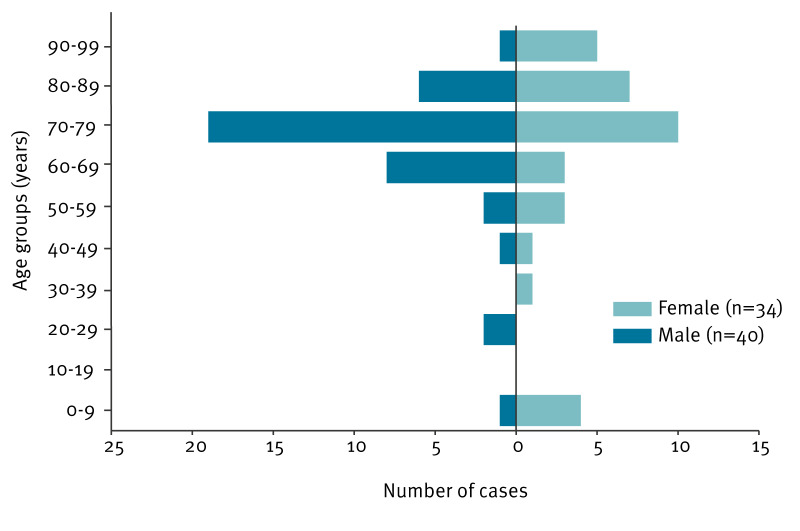
Distribution of cases of *Serratia marcescens* complex ct755 infection, by age group and sex, Norway, June 2021–February 2023 (n = 74)

Median age of cases was 76 years and 46% were female (Figure 3 and Table). The majority of cases had underlying conditions and a history of multiple hospital admissions. Among the 74 cases there were 19 deaths. For five of these, the infection was considered a strong contributing cause of death (median age: 86 years) and for six, it was a possible contributing cause of death (median age: 79 years). For the remaining eight deaths, infection with SMC ct755 was not considered a contributing cause of death (median age: 72 years).

**Table:** Characteristics of cases with *Serratia marcescens* complex ct755 infection, Norway, June 2021–February 2023 (n = 74)

Characteristics of cases		n	%
Sex (n = 74)			
Female		34	46
Male		40	54
Age (years)			
Median (IQR)		76 (65–80)	
Site of detection (n = 74)			
Hospital	Any ward	64	86
	Medical ward	35	47
	Surgical ward	12	16
	Cancer ward	7	9
	Intensive care unit	7	9
	Emergency unit	3	4
Outpatient clinic		3	4
Primary care / nursing home		3	4
Missing		4	5
SMC ct755 as cause of death^a^ (n = 19)			
Strongly contributing		5	26
Possibly contributing		6	32
Not contributing		8	42
Risk factors (n = 46)			
Peripheral venous catheter		43	93
Urinary catheter		22	48
Oxygen		22	48
Central venous catheter		17	37
Wounds		17	37
Ultrasound		16	35
Blood products		16	35
Surgery		15	33
Nasogastric tube		12	26
Nutritional drinks		10	22
Intubation		9	20
Tube feeding		9	20
Epidural/spinal catheter		7	15
Drain		7	15
Dialysis		6	13
Nebuliser		6	13
Laryngoscopy		5	11
Bronchoscopy/gastroscopy		5	11
Intermittent catheterisation		5	11
Parenteral nutrition		5	11
Stoma		4	9
Non-invasive pressure ventilation		3	7

IQR: interquartile range.

**^a ^** Clinical evaluation by treating physician.

Risk factors and exposure data (Table) were obtained from hospital records for 46 cases. Of these, 43 had a peripheral venous catheter – the only risk factor that was reported for more than half of the cases. The following most frequent risk factors were urinary catheter and oxygen treatment, each reported for 22 cases.

Date of hospital admission was available for 56 cases. For 30 of these, the outbreak strain ct755 was detected in a sample collected on the day of admission. Eleven of these 30 cases had been hospitalised in the 4 weeks before the current admission, nine had been hospitalised more than 4 weeks before, and six had not been hospitalised but had visited an outpatient clinic in the previous year. Importantly, four did not have any record of previous hospital stay or outpatient consultations. The remaining 26 cases had a positive sample taken between day 1 and day 91 of their hospital stay (Figure 2B).

The hospitalised patients were mainly admitted to surgical and medical wards. Seven were intensive care patients. Of note, three of the 74 cases were detected in the primary healthcare services and were not hospitalised (Table).

A total of 15 cases, nine female and six male, were interviewed about community exposures. Five of these were interviewed more than 2 months after they tested positive for ct755. No common exposure could be identified in terms of food products, medical products, cosmetic products, contact with healthcare facilities, contact with animals or other exposures.

### Descriptive epidemiology of ct281 (*Serratia bockelmannii*)

Among the 455 SMC isolates a total of 26 cases of SMC ct281 were detected between February 2022 and February 2023. We detected nine samples in blood cultures, five in respiratory samples, four in urine samples, three in wound samples and five in other materials; an epidemic curve for these cases, stratified by sample type, is appended in Supplementary Figure S1. Cases occurred in all four health regions. Median age was 67 years, 14 were female and 12 were male. Five cases were detected in primary healthcare. We collected data on exposures for 18 of the hospitalised cases. Again, peripheral venous catheter was the only risk factor that was reported for more than half of the cases. Suspected place of transmission was evaluated for 11 of the cases, of whom seven were reported to have been infected in the community and four presumably in the hospital.

### International inquiry

The two countries that responded to the inquiry published on EpiPulse did not report related outbreaks.

### Microbiological investigation

A total of 455 isolates belonging to the *S. marcescens* complex, collected from clinical samples in Norway between 1 January 2021 and 17 February 2023, were sequenced and analysed in the context of the outbreak investigation. This was not exhaustive, since an additional 18 isolates were identified only by use of a clone-specific PCR developed for the outbreak investigation and not subjected to WGS.

**Figure 4:**
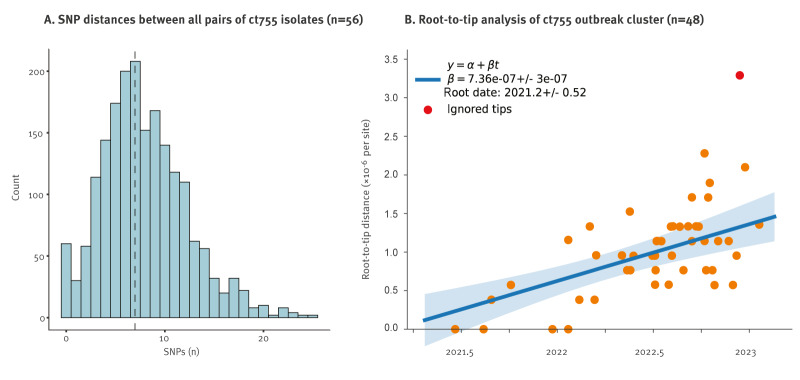
Within-outbreak diversity and temporal analyses, *Serratia marcescens* outbreak, Norway, June 2021 to February 2023

We used aligned whole genome assemblies as a starting point to generate a genome-wide SNP-based phylogenetic tree (Figure 1A). A total of 56 sequences were placed in the ct755 outbreak cluster; of these, 54 were assigned to ct755 using SeqSphere. The median genetic distance within the cluster was seven SNPs with all samples included (Figure 4A), and five SNPs when nanopore sequenced samples were excluded, demonstrating limited sequence diversity within the cluster. Samples from every health region were found in the ct755 cluster with no apparent link between genetic relatedness and geographical origin. This might suggest that a product contaminated by a common source has been distributed repeatedly across the country. In addition to the ct755 outbreak cluster, phylogenetic analyses also revealed the presence of two other relatively large clusters of isolates corresponding well with cgMLST classification as ct281 and ct833 (Figure 1A and B).

SNP: single nucleotide polymorphism.

Panel A: The median SNP-distance is marked with a dotted vertical line. Panel B: Graph created using TreeTime. To maximise accuracy, we excluded samples sequenced on the Oxford Nanopore platform from this analysis. The most recent common ancestor of all isolates was placed in March 2021 (confidence interval August 2020- July 2021). The ignored tip was automatically detected as an outlier with TreeTime. However, independent analyses using Tempest [26], which does not exclude outliers, reproduced an almost identical root date (2021.27, not shown).

We screened the first sequenced outbreak isolates belonging to ct755 and ct281 against all available *S. marcescens* genomes (n = 1,547) on the NCBI assembly database using Mash v.2.3 [27]. Subsequently, we quantified the SNP distances between the two outbreak isolates and their closest identified neighbour from the respective NCBI search by mapping reads from the outbreak isolates to the corresponding neighbour with Snippy. The assembly most closely related to ct755 (SO_2022_08_22_013) was identified as GCA_020884215_1_ASM2088421v1. The SNP distance was 4,036, demonstrating that they were not very closely related. For ct281, the closest identified relative was strain CM2019_254 (GCA_022439565_1_ASM2243956v1) with a SNP distance of 45. The strain CM2019_254 had been isolated from a horse guttural pouch biopsy in Australia in 2019.

To contextualise the Norwegian isolates, we used PopPUNK to cluster the assemblies together with 664 genomes from a recent global phylogenetic study of Serratia [4]. Based on these analyses, the Norwegian ct755 isolates were assigned to fastBAPS lineage 14 (level 3) as defined by Williams et al. [4]. In that article, lineage 14 was reported to be a non-pigmented lineage and suggested to be associated with various insect hosts. The assignment fits well with our observation that ct755 isolates were non-pigmented. We also placed ct755 in the context of Ono et al. [3] by downloading lineage-representative genomes from that study and matching ct755 against them using Mash [18]. These analyses demonstrated that ct755 belonged to *S. ureilytica* (lineage 12) in their framework, which was confirmed by independent classification against the GTDB.

To identify the time of origin of the outbreak source, we generated a dated ct755 phylogeny and performed a root-to-tip analysis using TreeTime [25] (Figure 4B). To maximise the accuracy of the analyses, we excluded samples sequenced on the Oxford Nanopore platform from this analysis, and SNPs were called by aligning fastq reads to a reference genome. The point of origin for the most recent common ancestor of all the ct755 isolates was estimated to lie between August 2020 and July 2021. As the first retrospectively identified isolate was collected in June 2021, we could further narrow down the interval to some time between August 2020 and June 2021.

## Discussion

We report a prolonged national outbreak with 74 cases of SMC ct755 involving 33 hospitals throughout Norway. The first identified case dates back to June 2021, and occasional cases were still being detected when the outbreak investigation was halted on 17 February 2023. The decision to stop the outbreak investigation was a difficult one, made when we observed a decline in new cases and based on an overall assessment balancing available resources and feasibility of identifying the source of the outbreak.

The observed characteristics of patients presenting with infection with outbreak strain ct755 did not stand out from what is usually observed with SMC infections. Most of the cases were 60 years and older and had extensive medical histories, often with severe underlying disease. Infection with ct755 was reported as strongly or possibly contributing cause of death among 11 cases. The investigation focused primarily on possible epidemiological links and common procedures or products used in hospitals as possible source of the outbreak. Review of hospital records of hospitalised cases showed no epidemiological links, and we did not identify unexpected common exposures.

Only a few cases were admitted to intensive care units at the time of detection. This reduced the suspicion of a nosocomial source somewhat, considering the vulnerability of intensive care patients and their massive exposure to medical products. Thirty cases had a positive sample with outbreak strain ct755 collected on the day of hospital admission, which indicates that the infection occurred before admission. However, a large proportion of these patients had a history of recent admissions, therefore previous exposure in the hospital setting was still a possibility. On the other hand, four of the hospitalised cases had no history of hospitalisation and had most probably acquired the infection outside the hospital environment. Also of note, the case-finding process identified three cases in the primary healthcare sector who were not admitted to hospital at all. Taken together, these observations pointed to an outbreak source primarily present in the community, or possibly a source present in both the community and in the hospital setting.

Establishing that the outbreak did not conform to a conventional nosocomial pattern and recognising the need to investigate potential community sources of the infections, consideration was given to additional investigative steps. This included weighting of required resources, the likelihood of successful detection of the outbreak source, and the estimated burden of disease caused by the outbreak. We had searched for possible community sources of the outbreak by interviewing cases, but the results of the interviews showed no epidemiological links or unexpected common exposures. Based on the literature, and having ruled out most other vehicles of transmission, our suspicion went towards a food product. The use of a standard hypothesis-generating trawling questionnaire for food exposures followed by a case–control study was considered but deemed an unsuitable method for this investigation of an opportunistic pathogen. A possible asymptomatic carrier state of unknown duration before clinical infection would complicate data collection and increase the potential for recall bias. Ultimately, the outbreak investigation was halted in February 2023 when the number of new cases declined. Local hospitals received a recommendation to continue monitoring incidence of SMC in clinical samples and to notify the NIPH in the event of an unusual increase. By August 2024, no new cases had been reported, and to our knowledge WGS is no longer performed on SMC isolates in the clinical laboratories.

During the outbreak investigation it became evident that ct755 was not the only clone of SMC that predominated in clinical samples during the study period from 1 January 2021 to 17 February 2023. A cluster of ct281 was also identified, involving hospitals throughout the country, and cases spanning in time from February 2022 until February 2023 when the investigation was halted (Cluster 2); see Supplementary Figure S1 for an epidemic curve. This cluster was subject to a parallel outbreak investigation. Some of the ct281 cases were identified in the primary healthcare services and were never hospitalised. Also, a majority of cases detected in hospitals were assumed to have been infected outside the hospital setting, thereby strengthening the hypothesis of a probable community-based origin. Further post-festum analysis of sequence data revealed an additional third cluster of closely related isolates, the majority belonging to ct833 (Cluster 3). Contrasting the clusters of ct755 and ct281, these isolates were collected over a limited time period of 8 weeks and, with two exceptions, originated from the same region. Cluster 3 was not subject to a national outbreak investigation.

Considering the population structure of all 455 sequenced SMC genomes, ct755 appears less unique than initially perceived and rather emerged as the largest and least diverse clone of a continuum. The three largest clusters together accounted for 21% of all samples. This has similarities to what was reported from Denmark in 2019 when WGS was performed on 701 clinical cases of *Campylobacter* infection: one large cluster persisted throughout the whole year and represented 12% of all studied *Campylobacter* cases [28]. The clonal nature of *Campylobacter* cases in Denmark had previously not been recognised. Interestingly, the predominant *Campylobacter* cluster was also detected in several isolates from chicken meat, and finally traced back to one slaughterhouse. We are not aware of any similar studies on SMC. The presence of SMC in food products and production facilities is increasingly well documented [29-32], but SMC is not considered to be enteropathogenic and food products are not tested for the presence of this low-virulent microbe. It is therefore possible that contamination of food products with SMC occurs with unknown frequency and goes undetected. We speculate that this may be of relevance to several previous publications that report extensive and prolonged outbreaks of *S. marcescens* without successful identification of the source despite intensive outbreak investigations [7,10,33].

The root-to-tip analysis of ct755 demonstrated that all isolates can be traced back to a single source, originating between the latter months of 2020 and spring 2021. The geographical spread of isolates did not correlate with genetic population structure, which suggests repeated national dispersal from a common unknown reservoir. This gives strength to the speculation that the outbreak was caused by a nationally distributed product but does not hint at what that product might be. We did not analyse environmental, medical, cosmetic or food samples, as we were not able to shortlist any suspected products. In Norway there is no dedicated laboratory resource for analysis or screening of such samples when a community-based source is suspected, a weakness that should be addressed.

## Conclusion

When investigating outbreaks caused by opportunistic pathogens such as those belonging to SMC, it is paramount to first assess whether there is a nosocomial source. If a nosocomial source can be ruled out, an assessment of the required resources and feasibility of further investigative steps should be considered carefully. For opportunistic bacteria, the search for a definitive source in the community may prove extremely difficult and require extensive resources. Our observations suggest that the presence of specific clones of SMC in clinical samples from different patients may be a relatively frequent phenomenon. Increasing use of WGS in clinical microbiology will continue to reveal the clonal nature of some bacteria and present dilemmas of interpretation to infection control personnel in public health.

## The Norwegian Serratia study group

Siv Anita Kvaal^3^, Lene Christin Olsen (ORCID;0000-0002-5869-4952)^4^, Torunn Gresdal Rønning (ORCID;0000-0003-4686-7614)^4^, Anette Skjærvik^4^, Randi Solheim^3^, Christina Gabrielsen Ås (ORCID;0000-0002-9605-3283)^4^, Iren Høyland Löhr (ORCID;0000-0002-8847-0044)^ 14,15^, Kirsten Gravningen (ORCID;0000-0002-8194-3208)^10, 20^, Silje Severine Sætre^10^, Karin Helmersen^10,21^ , Anne K. Steffensen^8^, Stephan A. Frye^8^, Jan Cato Holter (ORCID;0000-0003-1618-5022) ^8,20^, Hanne-Merete Eriksen-Volle^1^, Bjørn Iversen^1^, Turid M. Berglund^16^, Rune Jemtland^16^, Raymond Ludvigsen^17^, Bodil Weng^17^, Torunn Annie Pedersen (ORCID;0000-0002-9593-8119)^18^, Anna Kaarina Pöntinen (ORCID;0000-0002-3160-2042)^ 18,19^

Collaborator-specific affiliations:

14. Department of Medical Microbiology, Stavanger University Hospital, Stavanger, Norway

15. Department of Clinical Science, Faculty of Medicine, University of Bergen, Bergen, Norway

16. The Norwegian Food Safety Authority

17. The Norwegian Medicines Agency

18. Norwegian National Advisory Unit on Detection of Antimicrobial Resistance, Department of Microbiology and Infection Control, University Hospital of North Norway, Tromsø, Norway

19. Department of Biostatistics, Faculty of Medicine, University of Oslo, Oslo, Norway

20. Institute of Clinical Medicine, University of Oslo, Oslo, Norway

21. Department of Clinical Molecular Biology (EpiGen), Akershus University Hospital and University of Oslo, Lørenskog, Norway

## Supplementary Data

291000 Supplement
