# Supplementary material for: A national outbreak of Serratia marcescens complex: investigation reveals genomic population structure but no source, Norway, June 2021 to February 2023
Source: Euro Surveill. 2025 Feb 6;30(5):2400291. doi: 10.2807/1560-7917.ES.2025.30.5.2400291 (PMC11803742; doi:10.2807/1560-7917.ES.2025.30.5.2400291)
Supplement: Supplement [file 24-00291_TAXT_Supplement.pdf]

Supplementary data

This supplementary material is hosted by Eurosurveillance as supporting information alongside the article “A national outbreak of Serratia marcescens in Norway, June 2021 – February 2023: Investigation reveals genomic population structure but no source”, on behalf of the authors, who remain responsible for the accuracy and appropriateness of the content. The same standards for ethics, copyright, attributions and permissions as for the article apply. Supplements are not edited by Eurosurveillance and the journal is not responsible for the maintenance of any links or email addresses provided therein.

Fig. S1

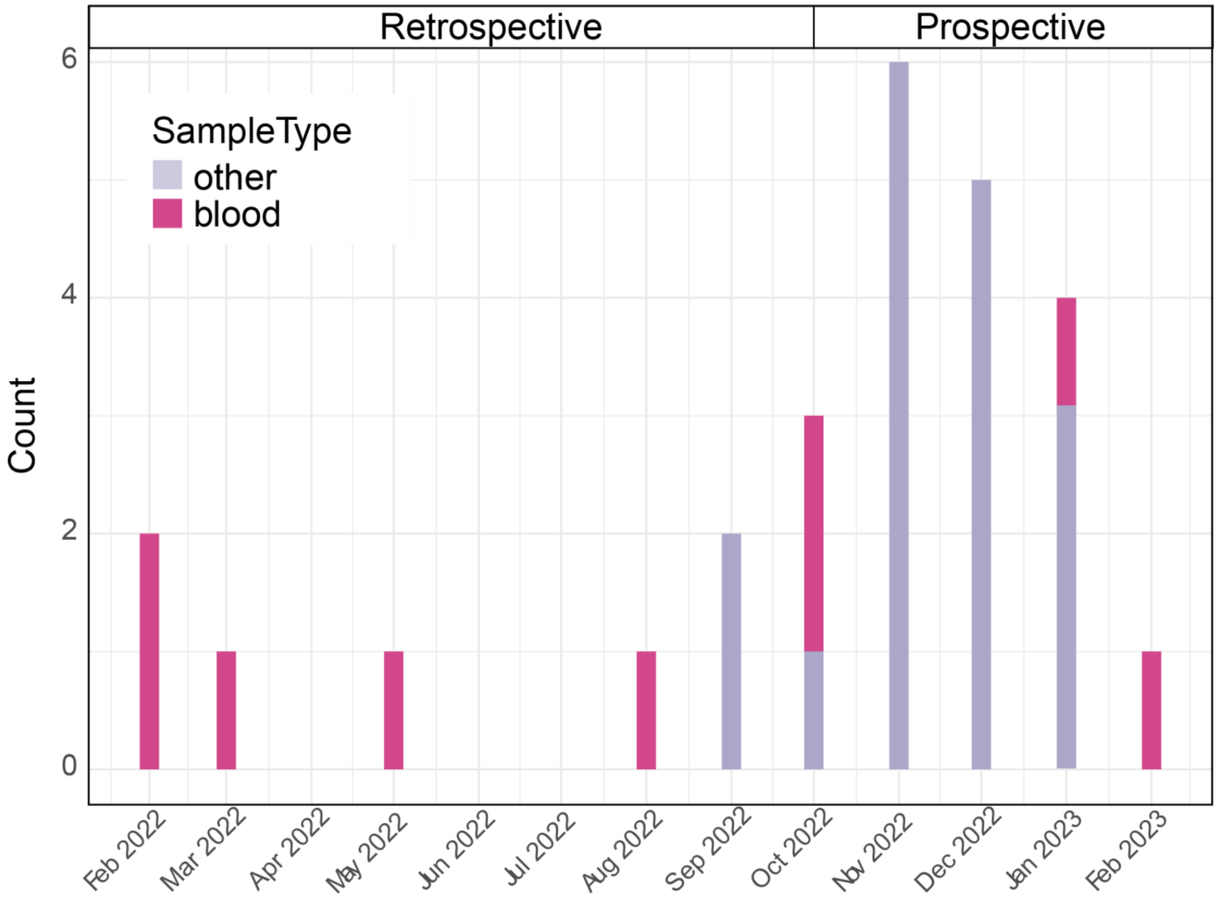

Fig. S1 Epidemic curve for cases of *Serratia marcescens* ct281 (n=26) stratified by sample type, Norway, February 2022 - February 2023.

Clone-specific PCR for *S. marcescens* ct755

A clone-specific multiplex real-time PCR was developed at the Department of Medical Microbiology, St. Olavs hospital, Trondheim University Hospital, for rapid detection of *S. marcescens* ct755. The PCR included an internal control with primers and probes targeting a species-specific site within the 16S rRNA gene (designed by Iwaya *et al.* 2005[1]) in addition to two strain-specific targets (*ammA*, *ammB*). The strain-specific primers and probes were designed using the IDT PrimerQuest™ tool based on results from a pangenome analysis using Roary[2]. The pangenome analysis included previously sequenced *S. marcescens* strains of ct755 (n=7) and other complex types (n=19) as well as a random selection of reference genomes downloaded from GenBank (n=109). The PCR mix contained 12 µM and 8 µM of primers and probes accordingly, in '2x PerfeCTa MultiPlex qPCR SuperMix, UNG' (Quantabio). The PCR program included UNG incubation (45 °C for 5 min), initial denaturation (95 °C for 3 min) and 35 cycles of 95 °C for 10 sec followed by 58 °C for 30 sec.

Table S1: Primer and probe sequences for multiplex real-time PCR for the detection of *S. marcescens* ct755.

Gene	Primer/probe	Sequence (5' -> 3')	5'mod	3'quencher	PCR-product size (bp)
16S rRNA	Forward	GGTGAGCTTAATACGTTCATCAATTG			179
	Reverse	GCAGTTCCCAGGTTGAGCC			
	Probe	TGCGCTTTACGCCCAGTAATTCCGA	FAM	BBQ	
ammA	Forward	GGCAACTCTAGGTATGGCTTAT			107
	Reverse	GTGAGTAGCGAAGCTCCAAA			
	Probe	ATCGCTCTTGACTCTTGGCGTGAA	VIC	MGBNFQ	
ammB	Forward	CTTAAGGACGAGACGGTTTCA			122
	Reverse	TCGGCGCCAAACGTATAA			
	Probe	CTCAGCCCAAGCAAGGTCATCATT	JUN	QSY	

References (Supplementary data only):

1. Iwaya A, Nakagawa S, Iwakura N, et al. Rapid and quantitative detection of blood Serratia marcescens by a real-time PCR assay: its clinical application and evaluation in a mouse infection model. *FEMS Microbiol Lett*. Jul 15 2005;248(2):163-70. doi:10.1016/j.femsle.2005.05.041
2. Page AJ, Cummins CA, Hunt M, et al. Roary: rapid large-scale prokaryote pan genome analysis. *Bioinformatics (Oxford, England)*. Nov 15 2015;31(22):3691-3. doi:10.1093/bioinformatics/btv421
